# Quinine in Cholera Infantum

**Published:** 1851-10

**Authors:** 


### QUININE IN CHOLERA INFANTUM.

We give among our selections, the experience of a physician of Tennessee in the use of sulphate of quinine in cholera infantum. Some years ago, we called the attention of our professional brethren to the same practice, observation having convinced us that this complaint of children was, in many cases, a form of intermittent fever. It will often be found on watching the progress of the case that it is paroxysmal, and when this feature manifests itself quinine is clearly indicated and is entirely efficacious. It is an interesting fact that cholera infantum appears to be on the decline in all parts of our country. Twenty-five years ago one of the most troublesome and fatal diseases among children, it is now but little dreaded. Whether this subsidence is but temporary, and after a time the complaint is to return with its former severity, is a question to which it would be difficult to give a satisfactory answer.

